# Deep learning automatically distinguishes myocarditis patients from normal subjects based on MRI

**DOI:** 10.1007/s10554-024-03284-8

**Published:** 2024-11-07

**Authors:** Cosmin-Andrei Hatfaludi, Aurelian Roșca, Andreea Bianca Popescu, Teodora Chitiboi, Puneet Sharma, Theodora Benedek, Lucian Mihai Itu

**Affiliations:** 1 Advanta, Siemens SRL, 15 Noiembrie Bvd, Brasov, 500097 Romania; 2 Automation and Information Technology, Transilvania University of Brasov, Mihai Viteazu nr. 5, Brasov, 5000174 Romania; 3 Cardiology Department, Emergency Clinical County Hospital of Târgu Mures, Târgu Mures, 540136 Romania; 4 Center of Advanced Research in Multimodality Cardiac Imaging, CardioMed Medical Center, Târgu Mures, 540124 Romania; 5 Siemens Healthcare GmbH, Lindenplatz 2, 20099 Hamburg, Germany; 6 Siemens Healthineers, Princeton, NJ 08540 USA; 7 Cardiology Department, “George Emil Palade” University of Medicine, Pharmacy, Science and Technology of Târgu Mures, Târgu Mures, 540139 Romania

**Keywords:** Few-shot learning, Deep neural networks, Medical imaging, CMRI, Myocarditis

## Abstract

Myocarditis, characterized by inflammation of the myocardial tissue, presents substantial risks to cardiovascular functionality, potentially precipitating critical outcomes including heart failure and arrhythmias. This investigation primarily aims to identify the optimal cardiovascular magnetic resonance imaging (CMRI) views for distinguishing between normal and myocarditis cases, using deep learning (DL) methodologies. Analyzing CMRI data from a cohort of 269 individuals, with 231 confirmed myocarditis cases and 38 as control participants, we implemented an innovative DL framework to facilitate the automated detection of myocarditis. Our approach was divided into single-frame and multi-frame analyses to evaluate different views and types of acquisitions for optimal diagnostic accuracy. The results demonstrated a weighted accuracy of 96.9%, with the highest accuracy achieved using the late gadolinium enhancement (LGE) 2-chamber view, underscoring the potential of DL in distinguishing myocarditis from normal cases on CMRI data.

## Introduction

Myocarditis, characterized by inflammation of the cardiac muscle, has implications on both the myocardial contractile function and the electrophysiological properties of the heart, potentially leading to heart failure and arrhythmias, respectively [1]. This condition’s etiology is multifaceted, encompassing infectious agents (for instance, viral pathogens like COVID-19 and parvovirus) [2], systemic inflammatory and autoimmune disorders, as well as adverse drug reactions. Clinical manifestations of myocarditis commonly include thoracic pain, lethargy, and dyspnea [3]. It is imperative for individuals presenting with symptoms suggestive of myocarditis to obtain prompt cardiological consultation to facilitate early detection and management. In cases of severe myocarditis, endomyocardial biopsy, a diagnostic intervention, is advocated to substantiate the diagnosis and inform therapeutic strategies [4].

Management of myocarditis includes a range of supportive measures, therapeutic interventions for symptomatic heart failure, administration of antimicrobial agents against identified infectious pathogens, and the use of immunosuppressive therapy in cases of severe inflammatory responses [5]. Timely diagnosis and the immediate commencement of treatment are crucial in significantly reducing the associated morbidity and mortality.

Non-invasive cardiac imaging, particularly CMRI [6], plays a vital role in confirming the diagnosis of myocarditis. In their study on myocarditis, the authors [7] underline the necessity of utilizing the Lake Louise Criteria (LLC) in CMRI, which involves analyzing cine, T2-weighted black blood, and LGE images to accurately diagnose the condition. However, the interpretation of CMRI is heavily reliant on expert analysis, which is both labor-intensive and susceptible to operator bias. To mitigate these challenges, the development of automated diagnostic systems utilizing advanced machine learning and data mining algorithms has been proposed. These systems are designed to efficiently address medical image classification challenges, thereby enhancing diagnostic accuracy and reducing subjectivity [8]. These technologies can be utilized within reporting workflows to automatically screen images, which helps in saving time for physicians, minimizing the incidence of errors, and improving the accuracy of diagnoses.

DL represents a category of machine learning algorithms characterized by the utilization of multiple layers to extract more abstract and advanced features from the raw input data [9]. In this study, we adopt the prototypical networks [10] approach for Few-Shot Learning (FSL). Prototypical networks operate by learning a metric space where each class is represented by a prototype, which is the mean of the examples in the embedding space. Classification is performed by computing the distance between the embedded query points and these class prototypes, enabling effective generalization to new classes with only a few examples. This approach simplifies the model’s inductive bias, making it particularly suited to the limited-data regime characteristic of FSL.

In the field of myocardial disease (MCD) diagnosis from CMRI, recent years have seen significant advancements through the use of DL techniques, as evidenced by various studies [3, 11, 12]. Sharifrazi et al. [3] introduced the Convolutional Neural Network-Clustering (CNN-KCL) model, specifically designed for MCD detection using CMRI images, with testing conducted on the Z-Alizadeh [3] dataset. This model incorporates a comprehensive approach by analyzing CINE-segmented images in both long axis (LAX) and short axis (SAX) views, Pre-contrast T2-weighted (TIRM) images in LAX and SAX views, T1-Weighted relative images pre-contrast and post-contrast in axial views of the myocardium, and LGE high-resolution phase sensitive inversion-recovery (PSIR) sequences in SAX and LAX views. The integration of these diverse views through a 2D-CNN with k-means clustering results in an impressive accuracy of 97.41%. Another noteworthy contribution by Shoeibi et al. [11] involved the application of the cycle-GAN method alongside various pre-trained models for MCD diagnosis, also utilizing the Z-Alizadeh dataset. The key innovation here was the use of cycle-GAN in preprocessing to generate synthetic CMRI images, which were then processed through different pre-trained models. Among these, the EfficientNet V2 method stood out, achieving an accuracy of 99.33%. Moravvej et al. [12] explored a different avenue by introducing deep reinforcement learning (RL) for MCD detection, presenting the RLMD-PA method for diagnosing myocarditis using CMRI images from the Z-Alizadeh dataset. Furthermore, the study examined various optimization methods to improve both the accuracy and efficiency of MCD diagnosis.

In this study, we introduce a DL approach designed to automate the detection of myocarditis from CMRI. Our methodology evaluates multiple CMR image sequences to ascertain which produces the best separation between normal and myocarditis cases. Through a comparative analysis of different imaging views of the heart (short and long axis), we aim to assess the complementary contribution of different views for myocarditis detection. Additionally, we compare two distinct techniques: FSL and classical binary classification, to determine which approach offers superior diagnostic precision. This investigation is critical for refining diagnostic precision and could significantly impact clinical decision-making by providing insights into the optimal CMRI view for diagnosing myocarditis, thereby enhancing patient care and treatment outcomes.

The remainder of this article is organized as follows. We start by discussing the available data and the model architecture (Materials and methods), followed by empirical results (Results) and a discussion and conclusions section (Discussions and conclusions).

## Materials and methods

### Dataset

#### Study design

This was a single-center, retrospective study that was carried out at the Center of Advanced Research in Multimodality Cardiac Imaging, Cardio-Med Medical Center, Târgu-Mureș, Romania. The study complied with the Declaration of Helsinki for investigation in human beings. The study protocol was approved by the local ethics committee and each patient signed an informed consent form before the enrolment in the study.

#### Study population

Patients at least 18 years old, with atypical angina, dyspnoea and fatigue are indicated for performing a CMRI. Further inclusion criteria were: history of cold/flu in the last 2–3 months, changes on the electrocardiogram (sinus tachycardia, where T negative diffuse), fever and chills. Patients were excluded if they were unable to provide informed consent, and if they presented with myocardial ischemic injury (history of myocardial infarction STEMI and NON-STEMI), autoimmune diseases and infiltrative diseases at the myocardial level (amyloidosis). The data was collected between August 2021 and September 2023. A total of 269 patients were included in the study, of whom 231 had myocarditis, while the remaining 38 were control subjects. This binary label was derived based on the clinical report and represents the final clinical consensus. For this reason, a multi-observer study could not be performed. Patients from the control group have no other cardiac disease. However, the number of views per patient differs and some patients are missing specific views, which can result in a small difference in the number of samples employed during training for specific views.

#### Procedure protocol

Each patient included in the study was subjected to an CMRI examination with consent. Based on the obtained results, patients were divided into the two study groups. To perform the cardiac scenarios, we used CMRI acquired with a Magnetom Aera 1.5 T scanner (Siemens Healthineers AG, Erlangen, Germany).

A standard cardiac MR protocol was employed including cine balanced steady-state free precession (bSSFP) short and long axis views, T2-weighted acquisitions, and LGE in LAX 2-chamber and 4-chamber views. For the LGE acquisition a bolus of gadolinium-based contrast agent (Gadovist) was injected at a rate of 4 ml/s. Ten minutes after injection a PSIR sequence was acquired at the same LAX positions as the cine bSSFP to detect LGE.

**Fig. 1:**
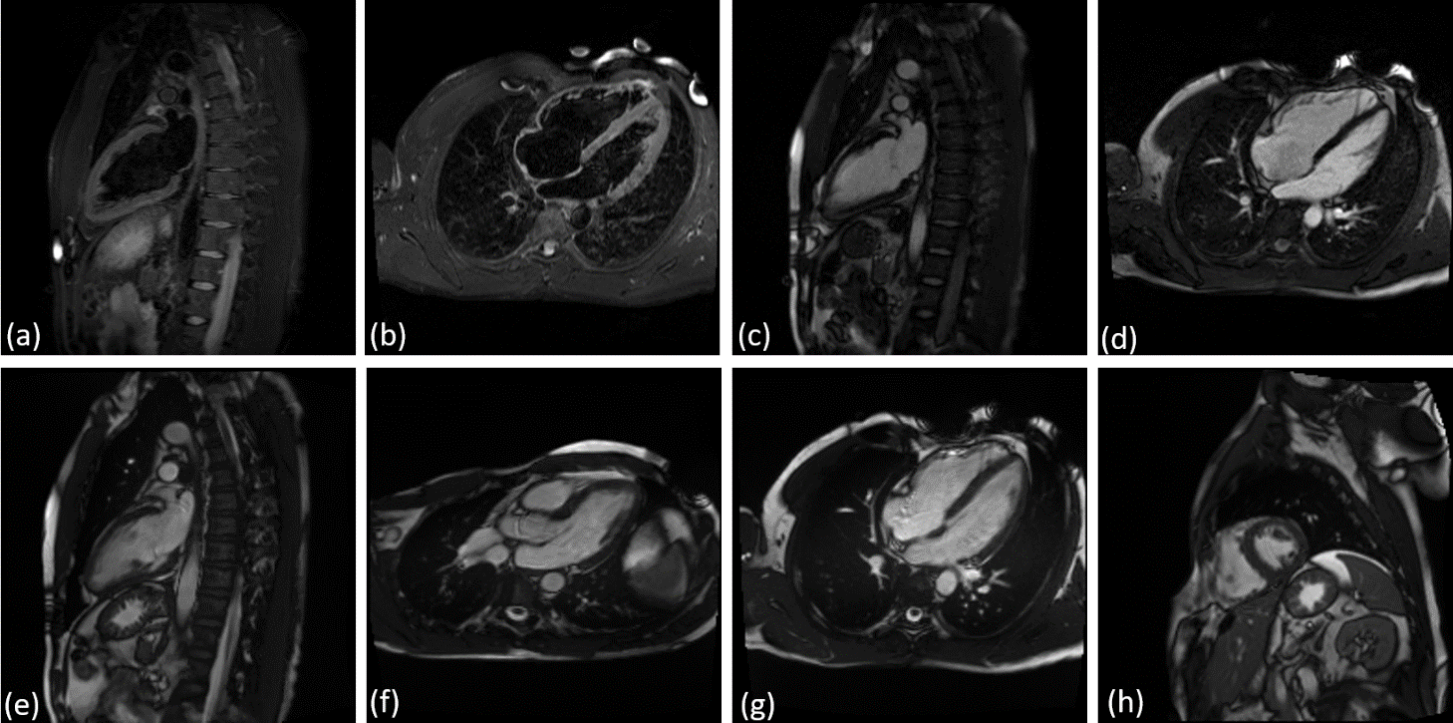
Views considered for the detection of myocarditis from CMRI: **(a)** T2 weighted 2-chamber view, **(b)** T2 weighted 4-chamber view, **(c)** LGE 2-chamber view, **(d)** LGE 4-chamber view, **(e)** cine bSSFP 2-chamber view, **(f)** cine bSSFP 3-chamber view, **(g)** cine bSSFP 4-chamber view, **(h)** cine bSSFP SAX stack. The middle frame is displayed for multi frame views, and for the multi-slice multi-frame view, we used the middle slice and the middle frame

Following the suggestion of the clinical experts in the study, the following CMRI acquisitions were considered for the detection of myocarditis:

- PSIR LGE in 2-chamber and 4-chamber views (single frame).
- T2-weighted 2-chamber and 4-chamber views (single frame).
- Cine bSSFP in LAX 2-chamber, 3-chamber, and 4-chamber views (multi-frame).
- A SAX stack of cine bSSFP (x to y numbers) slices covering the left ventricle (multi-frame, mult-slice).

The term clinical expert refers to physicians who evaluate patients and establish the diagnosis of myocarditis. These clinicians have over 10 years of clinical experience in the field of cardiac pathology (including myocarditis). Additionally, for the imaging review, a senior radiologist with over 10 years of experience was involved, practicing as a radiologist for more than 40 years.

Figure 1 displays examples of all image acquisitions and views considered in the analysis. For the multi-frame views, we display the middle frame and for the multi-slice multi-frame view we depict the middle frame of the middle slice. We adapted a DL approach for each of the single frame, multi-frame, and multi-slice type of data to perform myocarditis vs. normal classification.

### Data pre-processing

**Fig. 2:**
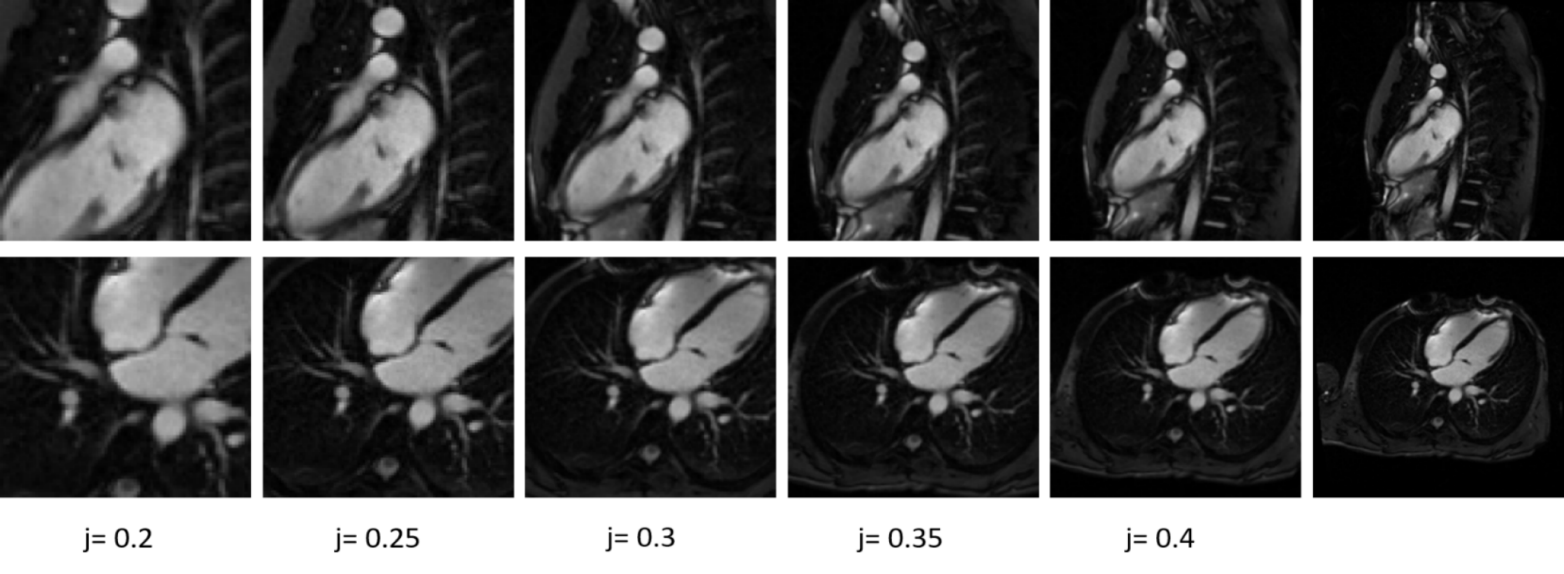
Visual illustration using different increments of k for LGE 2-chamber view and LGE 4-chamber view

All images were normalized using z-score normalization [13]. We computed the mean and standard deviation for the training data present in each view, respectively we normalized each image using the mean and the standard deviation obtained for that specific view. To potentially enhance the neural network’s performance by focusing on the region of interest, the images were cropped. Given the central location of myocardium in the images, the cropping procedure was implemented as follows: we retained the central 50% of the image in each dimension plus a proportional segment (j) of the image size: ($\:\frac{imag{e}_{size}}{2})+(j*imag{e}_{size})$. Parameter j was set at 0.5 (non-cropped images) and at increments of 0.05 ranging from 0.4 to 0.2. Details are included in the following subsections (see Fig. 2).

To evaluate the performance we computed the weighted accuracy [14]:

1 $$\:weighted\:accuracy=\:0.5*(\frac{TP}{TP+FN}+\:\frac{TN}{TN+FP})$$

Even though the weights are equal (0.5), the use of weighted accuracy ensures a balanced consideration of both sensitivity (true positive rate) and specificity (true negative rate). This approach is particularly important in medical imaging studies where dataset imbalance is common. and it helps in maintaining consistency and clarity in reporting performance metrics. In our study, while the weights are equal, using weighted accuracy provides a framework that can easily adapt to future studies with different class distributions.

For the models that showed the highest weighted accuracy, receiver operating characteristic (ROC) analysis was performed as outlined in [15], and the area under the curve (AUC) score was calculated [16]. The selection of the optimal threshold for each model was guided by the ROC curves, identifying the point that most closely approached the ideal (0,1) coordinate, consistent with the methodology recommended in [17]. The chosen threshold was then applied to obtain the results, which are reported using various performance metrics (weighted accuracy, sensitivity, specificity, PPV, NPV) [18].

The determination of the cut-off point closest to the coordinate (0,1) was achieved using the equation [19]:

2 $$\:ER\left(c\right)\:=\sqrt{(1-{Se\left(c\right))}^{2}+(1-{Sp\left(c\right))}^{2}}$$

Here, ER quantifies the shortest distance to the coordinate (0,1), *c* signifies the cut-off point, *Se* is sensitivity, and *Sp* represents specificity.

All the models were implemented using Python, specifically Pytorch [20]. All statistical analysis were also performed in python.

### Classification based on single frame images

The single-frame views were processed using a neural network architecture comprising six convolutional layers followed by a fully connected layer to generate the final output. The task was structured as a binary classification problem [21], and we adopted two distinct training strategies: a classical approach and a FSL approach. In the classic training paradigm, a sigmoid activation function [22] was used at the model’s output layer to obtain probabilistic predictions ranging from 0 to 1. Conversely, for the FSL strategy, we omitted the terminal fully connected layer. For this study we used prototypical networks implemented as in [10] for FSL.

In this study, given the constraints of a limited dataset, we used k-fold cross-validation [23] with k = 2, over 50 training epochs using the following procedure. First, a model was trained from scratch on K1 and evaluated on K2 for a total of 50 epochs. Second, the same model architecture was trained from scratch on K2 and tested on K1 for 50 epochs. This process ensured that both models were trained independently on independent subsets of the data, enabling their comparison.

To evaluate their performance, we computed the weighted accuracy for each epoch on the respective test set. At each epoch, we computed the performance of each model, and averaged the performance to obtain an overall score. We then select the best training epoch using this score. The defined score is described in the Data pre-processing section.

To preserve data integrity, the dataset was divided such that all CMRIs from a single patient were grouped into the same fold, preventing any patient’s data from being distributed across multiple folds. We also ensured a balanced distribution of normal and myocarditis cases in both folds to maintain class proportions across folds. The models were consistently trained using the Adam optimizer [24] with a learning rate maintained at 0.0001. For the last layer of the model, we have used a dropout function with a dropout rate of 0.5. As a loss function, for the classic models we have used the binary cross entropy (BCE) [25] and for FSL we have used the negative log-probability as described in [10]. The data augmentation process involved applying intensity perturbation, random rotation within ± 10 degrees, and zooming between 0.9x and 1.15x to enhance the variability of the training dataset.

### Classification based on multi frame images

**Fig. 3:**
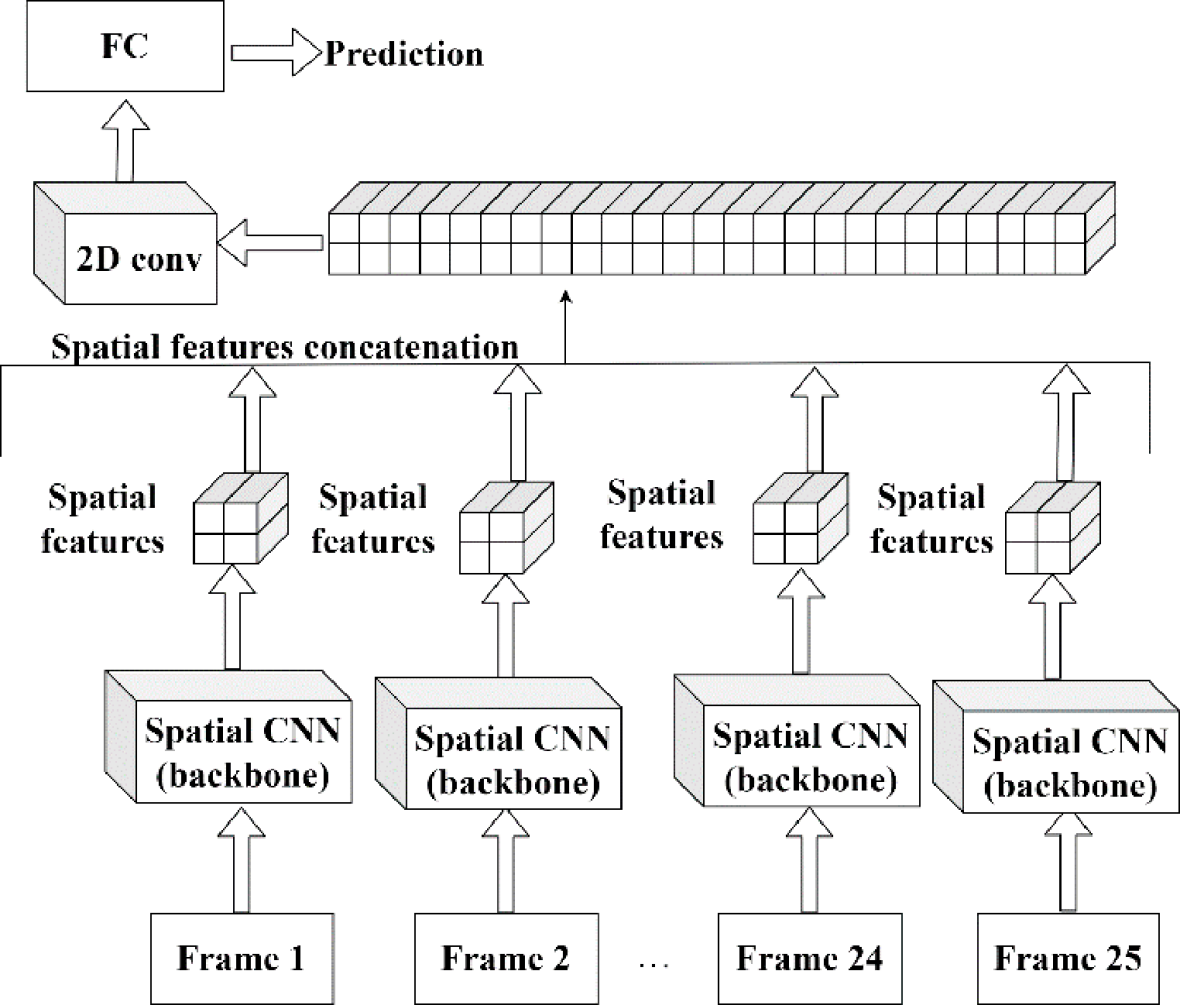
Illustration of the multi-frame processing approach using a spatial CNN backbone with six convolutional layers. This diagram depicts the process where the backbone is applied independently to each frame, followed by the concatenation of resulting feature maps from all frames. A subsequent single 2D convolutional layer is then utilized to extract and integrate both spatial and temporal features across the entire frame set

For processing the multi-frame views, our approach involved using a spatial CNN (backbone) with six convolutional layers. The backbone was applied to individual frames independently. The resulting feature maps from each frame were then concatenated. Subsequently, a single 2D convolutional layer was applied to this aggregated feature set. The goal of this layer is to extract both spatial and temporal features from all frames. An illustration of our approach is depicted in Fig. 3.

Similar to the single-frame views, the task was defined as a binary classification problem. For this, we employed two distinct methodologies, consistent with the ones previously described for single-frame view analysis. All multi frame views have a fixed number of frames (25).

**Fig. 4:**
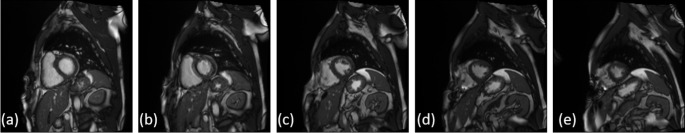
The five middle slices for the multi slice multi frame view (cine bSSFP SAX stack): (**a**) slice 5, (**b**) slice 6, (**c**) slice 7, (**d**) slice 8, (**e**) slice 9. The middle frame is depicted for all 5 slices

For the stack of cine bSSFP SAX slices, each slice was processed independently using the approach depicted in Fig. 3. The training of the model on this particular view was conducted under three different scenarios: first, by using the complete set of slices for each patient; second, by employing a subset of three contiguous slices centred around the middle slice, and third, by selecting a broader subset of five slices centred around the middle slice (see Fig. 4). For each scenario, we calculated the weighted accuracy to evaluate the model performance. Based on this outcome, the scenario with the highest accuracy was adopted for all subsequent experiments pertaining to this view.

### Post processing analyses

To analyze the features that the model is focusing on, we computed the saliency maps [26] for the models obtained on the views leading to the highest weighted accuracy. To obtain the saliency map for those models, we computed the derivative of the output with respect to the input for each individual model.

Additionally, we also used the best performing models to conduct a series of subgroup analysis based on the available patient characteristics.

## Results

### Population characteristics

Baseline patient characteristics are summarized in Table 1.

**Table 1:** Baseline patient characteristics and risk factors

Male	137 (51.89%)
Female	127 (48.1%)
Age (years)	54.46 ± 15.87 years
Origin	All European
Weight	75.01 ± 10.96 kg
Height	169.79 ± 7.13 cm
Diabetes	34 (12.84%)
Hypertension (HTA)	193 (73.1%)
Hypercholesterolemia	156 (59.09%)
Smoking history	71 (26.89%)
Previous Angina	15 (5.68%)
Forced expiratory volume (FEVS)	47.47 ± 4.58%

### Cine bSSFP SAX stack results

The results obtained using the cine bSSFP SAX stack for classification are reported in Table 2. The best results for this view are obtained using three slices. This is to be expected since the myocardium is typically best visible in the middle slices. Hence, for all other experiments and results presented herein for this view, we have used a fixed number of slices equal to three.

**Table 2:** Weighted accuracy obtained for the cine bSSFP SAX stack, for different number of slices selected as input for the classification network

View	Classic	FSL	Classic	FSL	Classic	FSL
Number of slices	All		3		5	
Weighted accuracy [%]	64.2	61.9	67.2	67.5	65	67.3

### All

The weighted accuracy computed for all the previously described models are reported in Table 3. The best results were obtained for PSIR LGE images in 2-chamber and 4-chamber views. On both views the model obtained an accuracy greater than 90%. On the single frame views the models achieved higher overall performance than on the multi frame views. Cropping the images using increments of j improved the performance for some views (e.g. T2 weighted 4-chamber view, cine bSSFP 2-chamber view), but for the best-performing views, the performance did not increase, and using the original images yielded the best results.

**Table 3:** Weighted accuracy obtained for the single- and multi-frame views considered in this study

View	Classic	FSL	Classic	FSL	Classic	FSL	Classic	FSL	Classic	FSL	Classic	FSL
j	0.5 (original)		0.4		0.35		0.3		0.25		0.2	
T2 weighted 2-chamber view	67.2	**62.3**	56.7	62.3	66.8	61.3	63.5	59.2	63.2	57.5	**69.9**	57
T2 weighted 4-chamber view	69.0	72.0	69.9	72	**71.8**	72.6	66.3	**74.8**	65.1	71.1	64.5	72
**LGE 2-chamber view**	**93.0**	**96.9**	89.2	95.6	91.4	90.7	85.7	87	84.4	88.1	86.6	87.2
**LGE 4-chamber view**	**87.4**	**90.1**	84.8	87.6	85.6	88.5	85.4	88.3	87.2	87	85.7	84.8
cine bSSFP 2-chamber view	66.3	67.3	68.2	72.9	66.1	75.2	70.7	73.5	67.9	**76.8**	**75**	75.8
cine bSSFP 3-chamber view	56.3	56.0	**57.6**	53.6	54.4	54.9	51.7	53.4	50	52.1	52.1	**56.2**
cine bSSFP 4-chamber view	56.3	63.0	62.6	**64.3**	**64.4**	60.2	59.2	62.6	63	63.1	56.7	61.7
cine bSSFP SAX stack (3 slices)	**67.2**	67.5	64.7	69.6	64.2	70.3	62.2	70.2	63.2	69.6	61.1	**71.2**

Table 4 displays the number of samples with myocarditis and without myocarditis for each view in our dataset. Although the patient number is the same, for some patients certain views are missing views.

**Table 4:** The numbers of samples for each view

View	Normal	Myocarditis
T2 weighted 2-chamber view	38	225
T2 weighted 4-chamber view	38	225
**LGE 2-chamber view**	38	226
**LGE 4-chamber view**	38	226
cine bSSFP 2-chamber view	38	227
cine bSSFP 3-chamber view	9	161
cine bSSFP 4-chamber view	38	227
cine bSSFP SAX stack (3 slices)	38	226

**Fig. 5:**
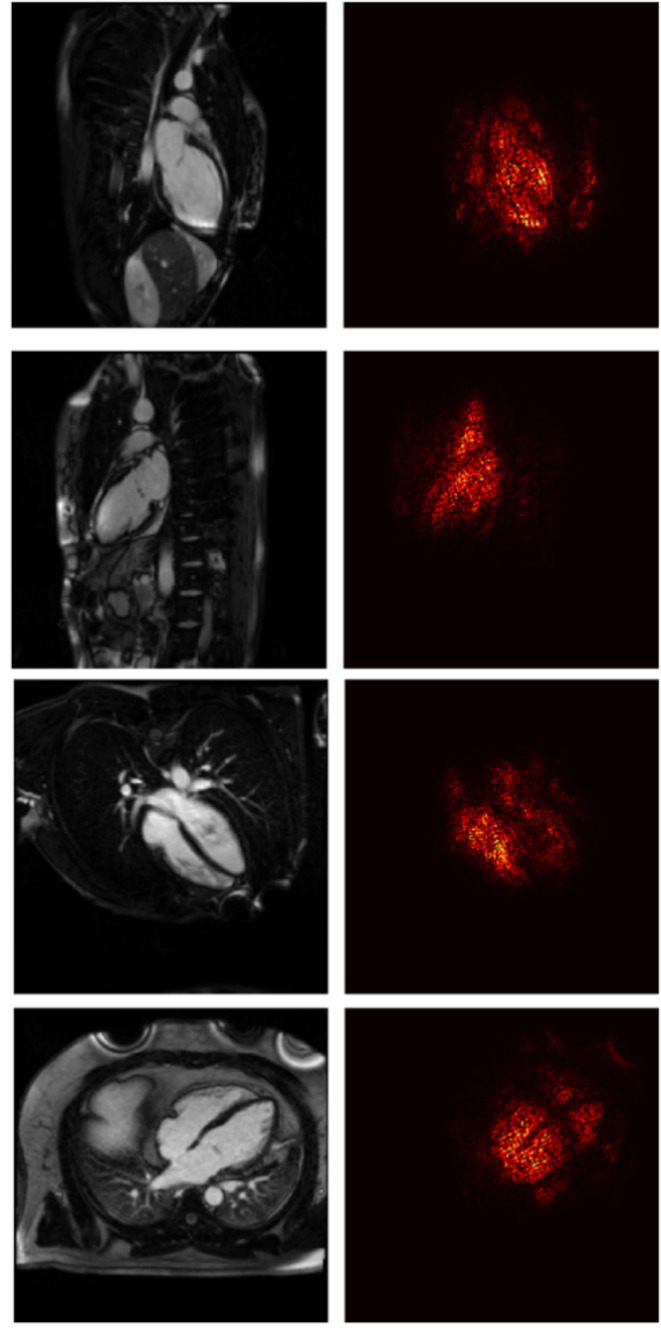
Two sample saliency maps are shown for the LGE 2-chamber view (top row) and the LGE 4-chamber view (bottom row). For each view, the saliency maps are presented with the control subject on the top and the myocarditis case on the bottom

For the saliency analysis, we considered the two best-performing models and views. Sample saliency map for both the LGE 2-chamber and 4-chamber views are displayed in Fig. 4. The top two images in Fig. 5 represent the 2-chamber view, where the model predominantly examines the myocardial region to perform predictions. Similarly, the bottom two images represent the 4-chamber view, with the model focusing primarily on the myocardial region for the predictions.

### Statistical analysis

**Fig. 6:**
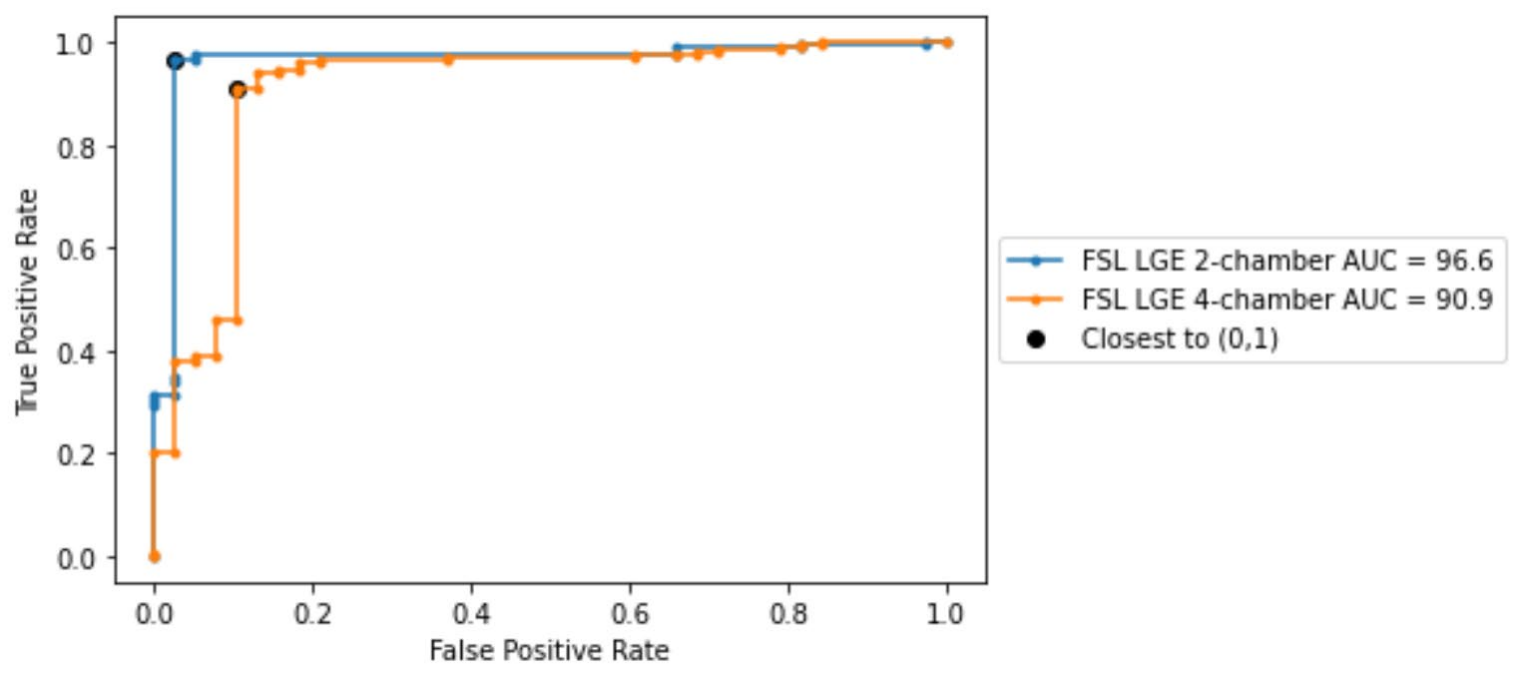
ROC curvers obtained for the best performing models, i.e., employing FSL on the LGE 2-chamber and LGE 4-chamber views respectively

In this subsection we further analyze the best performing models in terms of weighted accuracy. The ROC curves for both models are depicted in Fig. 6, alongside the AUC score. Both models reached AUC scores greater than 90% and the best results were obtained on LGE 2-chamber view. Using the thresholds derived from the ROC curves, we computed the performance metrics for both models (see Table 5). Given the already close to optimal performance, using the thresholds derived from the ROC curves did not further improve the model performance in terms of weighted accuracy. For the best two models, we have computed the DeLong test [27, 28]. The P-value between the models in Table 5 is 0.08, indicating that the difference between the models is statistically not significant.

**Table 5:** Performance metrics obtained for the best performing models

View	Accuracy	Weighted_Accuracy	Sensitivity	Specificity	PPV	NPV	AUC score
**LGE 2-chamber view**	96.9	96.9	96.4	97.3	99.5	82.2	96.5
**LGE 4-chamber view**	90.3	90	91.1	89.4	98	61.8	90.9

**Fig. 7:**
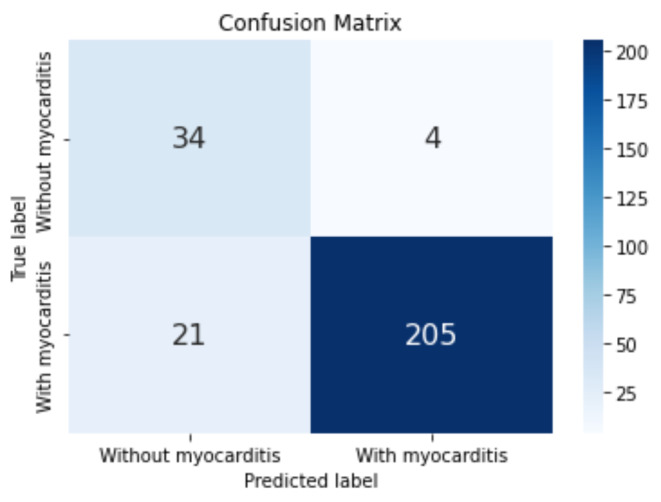
Confusion matrix obtained for the model corresponding to the LGE 2-chamber view-based classification.

The confusion matrix for the best model obtained on LGE 2-chamber view is depicted in Fig. 7: a single FN was obtained alongside eight FPs.

**Fig. 8:**
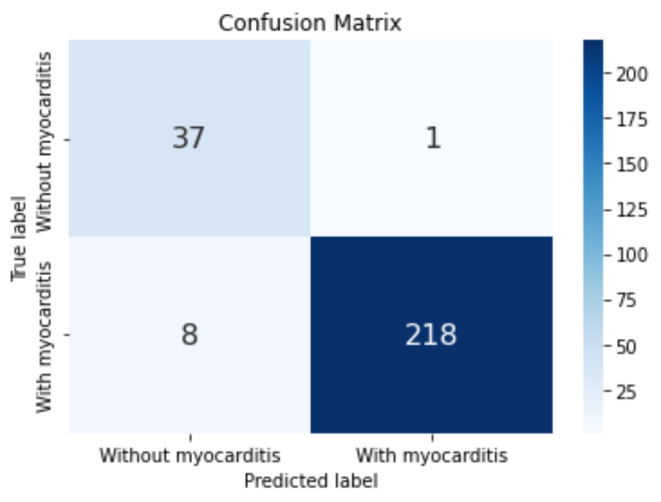
Confusion matrix obtained for the model corresponding to the LGE 4-chamber view classification

The confusion matrix for the best model obtained on LGE 4-chamber view is depicted in Fig. 8.

**Fig. 9:**
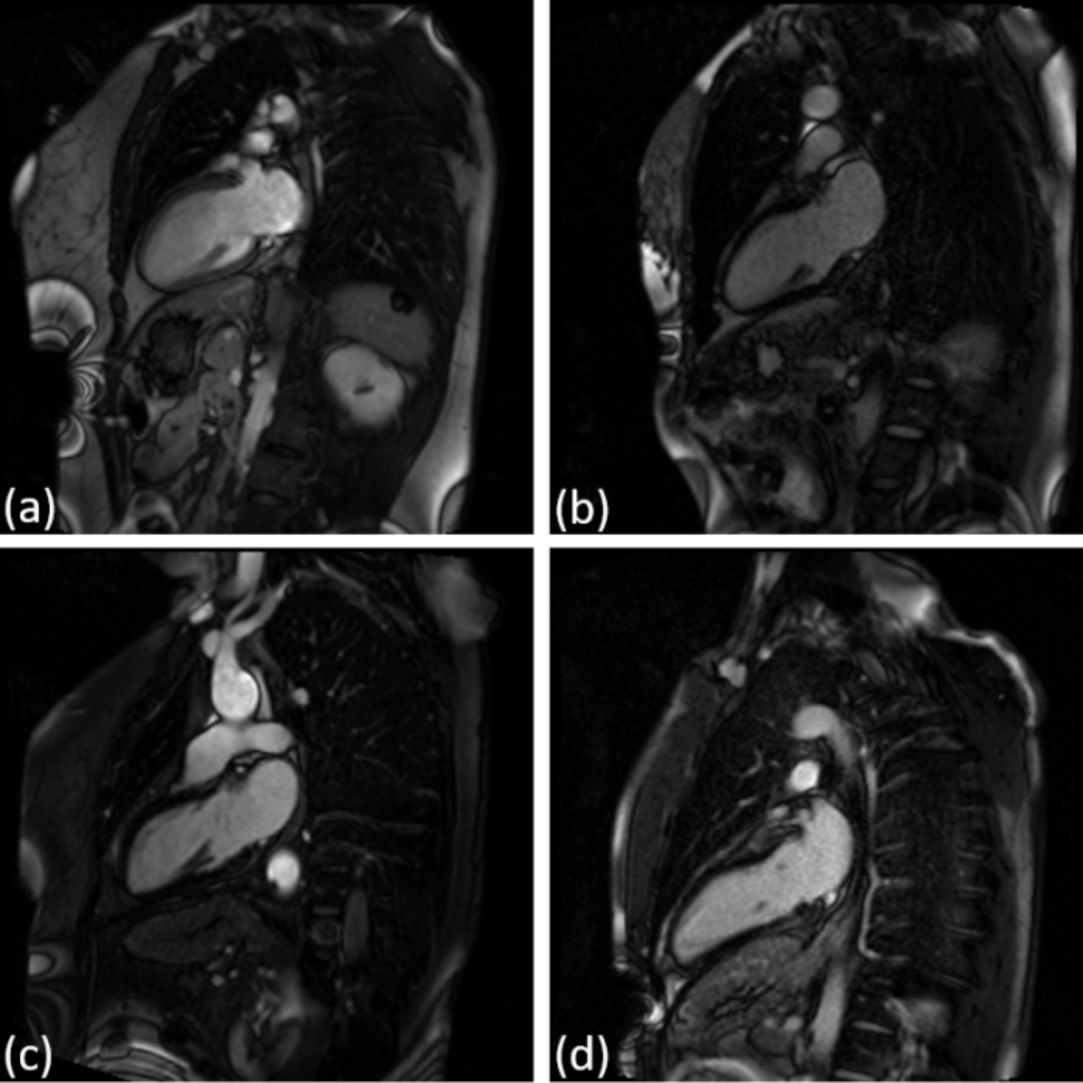
Four sample cases for the LGE 2-chamber view: (**a**) TP, (**b**) FP, (**c**) TN, and (**d**) FN

**Fig. 10:**
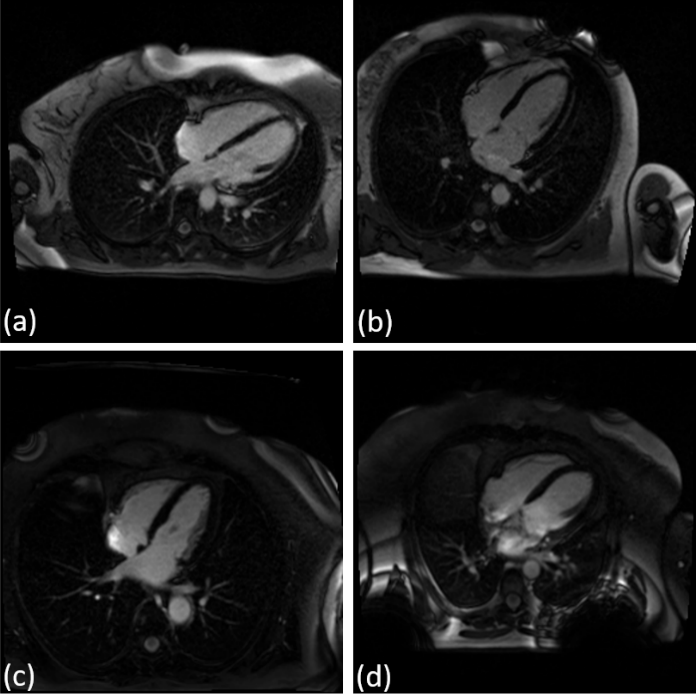
Four sample cases for the LGE 2-chamber view: (**a**) TP, (**b**) FP, (**c**) TN, and (**d**) FN

Figures 9 and 10 display four sample cases from the dataset: one true positive (TP), one true negative (TN), one false positive (FP), and one false negative (FN) for LGE 2-chamber view and LGE 4-chamber view.

For the age-based subgroup analysis, patients were divided into three equally sized bins. No significant variations in weighted accuracy were observed across the age intervals in the 2-chamber model. However, the 4-chamber model showed improved accuracy for patients older than 62 years.

For the weight-based subgroup analysis, the 2-chamber model demonstrated superior accuracy across all weight ranges, with the highest accuracy in individuals weighing over 79 kg. The slight decrease in accuracy for patients under 69 kg indicates sensitivity to patient physique.

In the diabetes subgroup analysis, both models exhibited high accuracy in diabetic patients. The 2-chamber model maintained consistent accuracy across diabetic and non-diabetic patients, while the 4-chamber model showed a notable decrease in non-diabetic patients.

For the HTA subgroup, the 2-chamber model consistently outperformed the 4-chamber model in both hypertensive and non-hypertensive patients.

For patients with hypercholesterolemia, the 2-chamber model achieved slightly higher accuracy than the 4-chamber model. No notable difference in performance was observed between patients with or without hypercholesterolemia.

Regarding smoking status, the 2-chamber model showed exceptional accuracy, particularly among smokers, indicating superior effectiveness in myocarditis detection across smoking statuses.

In the angina subgroup, both models performed better in patients with angina, likely due to specific imaging markers introduced by angina.

For LVEF, the 2-chamber model maintained high accuracy across different LVEF values, while the 4-chamber model performed better as LVEF increased.

Given the limited number of samples, the conclusions drawn above require confirmation on larger datasets.

**Fig. 11:**
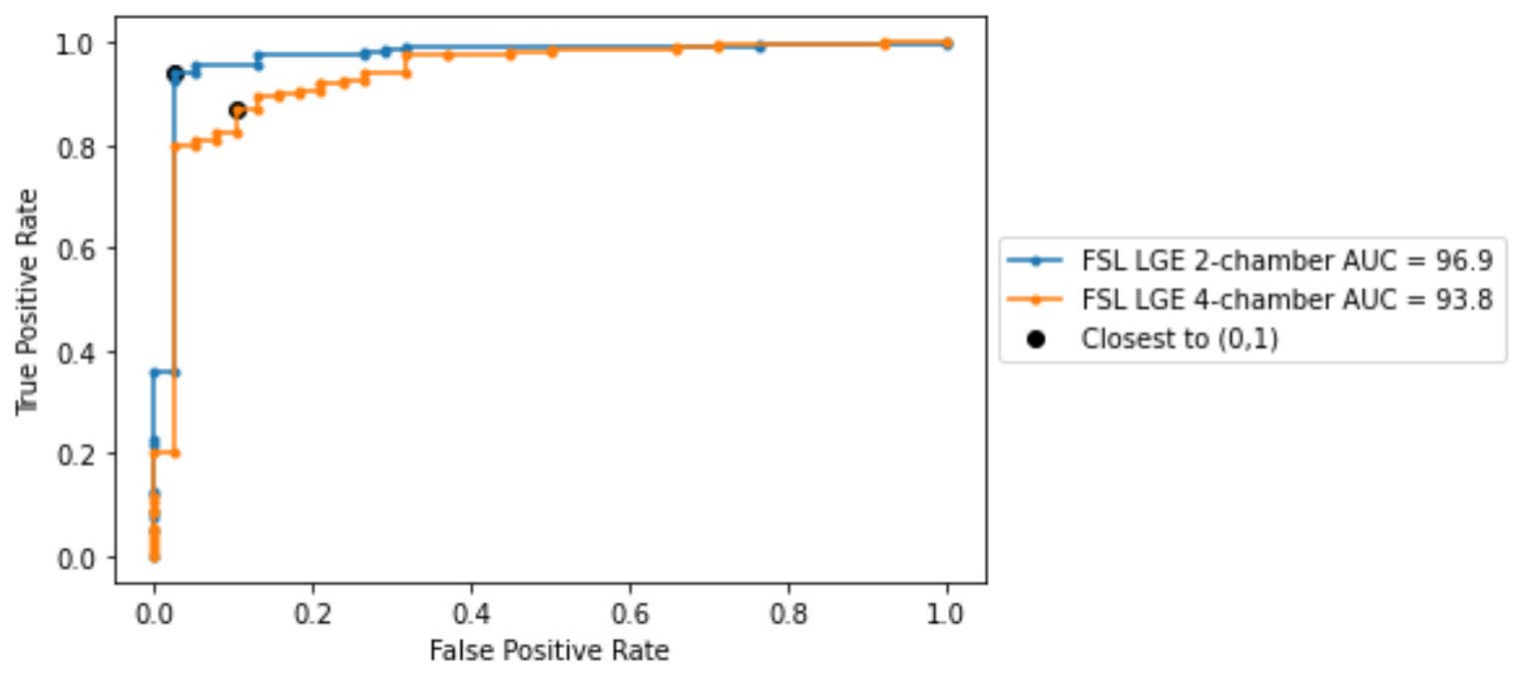
ROC curves obtained at the last epoch for the best performing models, i.e., employing FSL on the LGE 2-chamber and LGE 4-chamber views respectively

For the best performing models, we have included the metrics computed for the last epoch (Table 6) and the ROC curves (Fig. 11).

**Table 6:** Weighted accuracy of the myocarditis classification task obtained for patient subsets

Gender	2-chamber [%]	4-chamber [%]
Male	98.2	89.3
Female	95	90.5
Age > 62 years	96.7	96.7
Age > 48 years and Age ≤ 62 years	98.7	85.2
Age ≤ 48 years	97.2	89.4
Weight > 79 kg	100	94.3
Weight ≥ 69 kg and Weight ≤ 79 kg	97.5	93.2
Weight < 69 kg	94.2	86.6
Diabetes	96.8	98.4
No Diabetes	97	89.2
Hypertension (HTA)	97.9	92.9
No HTA	96.1	86.5
With hypercholesterolemia	97.4	91.4
Without hypercholesterolemia	96.9	89.6
Smoker	99.1	92
Non-smoker	95.8	89
Angina	100	96.4
No Angina	96.7	89.8
Left ventricular ejection fraction (LVEF) < 50%	97.9	85.4
LVEF ≥ 50%	97	90.7

**Table 7:** The metrics computed for the best performing models at the last epoch

View	Weighted Accuracy	Sensitivity	Specificity	AUC score	TP	TN	FP	FN
**LGE 2-chamber view**	94.9	94.2	97.3	96.9	209	37	1	17
**LGE 4-chamber view**	88.3	87.1	89.4	93.7	208	30	8	18

## Discussions

Comparative analyses demonstrated that the FSL approach outperformed the classical training method across the majority of the image types and image views, underscoring its efficacy. In the control group, which includes 38 cases, the artificial intelligence algorithm identifies 1 case as possibly positive for the diagnosis of myocarditis. The patient was in the 24–40 years range, and his history has identified a recent viral disease. However, the patient was clinically asymptomatic, and showed no changes in laboratory tests. CMRI imaging of the patient showed a small punctate change in the myocardium, indicating possible microvascular damage at this level without clinical significance. This fact proves that the algorithm developed during this study has a high detection rate of changes, even minor, occurring at the myocardial level.

Analyzing patients in the myocarditis group, we found that the artificial intelligence algorithm identified from a total of 231 cases, 8 cases, as being false negative. These false-negative results were likely caused by diffuse distribution of a small amount of fibrosis in the myocardium, with patients presenting with forms of self-limiting myocarditis. For some patients diagnosed with myocarditis it is possible that the fibrosis region is only visible in the 2-chamber or only in the 4-chamber orientation, further complicating the single image series-based classification. Another possible cause of these false negative results could also be the relatively small number of patients included in the group with myocarditis. Recent studies have proven that a larger number of patients included in the artificial analysis algorithm allows it to detect even those minor changes occurring in the myocardium [29, 30].

In the area of myocardial disease diagnosis based on CMRI, our study adopts an approach where each CMR image sequence is examined independently, a strategy that differs from those seen in prior studies like those by Sharifrazi et al. [3], Shoeibi et al. [11] and Moravvej et al. [12]. The approaches present in literature achieved an accuracy between 97.41 and 99.33% on the Z-Alizadeh dataset. Our model achieved a weighted accuracy of 96.9% for myocarditis detection on LGE 2-chamber view. While the results are slightly lower (0.5% difference), the results are not directly comparable. Our approach enabled a granular examination of how individual imaging views—ranging from T2-weighted acquisitions to LGE sequence—contribute distinctly to the accuracy and reliability of myocarditis detection. By evaluating the diagnostic performance of each view, our research not only identified the most effective sequences for myocarditis detection but also offered a rich, multi-faceted understanding of the disease’s radiological presentation. This contrasts with the aggregated view analysis in other studies, which, while effective in harnessing composite information, may overlook the unique diagnostic value embedded within each specific imaging angle. The saliency analysis, in particular, offers valuable insights into what the algorithm considers important when making decisions, focusing more on how the model prioritizes different areas of the images for diagnosis. This saliency analysis underscores our model’s reliance on features within the myocardial region and fosters a greater understanding of the patterns indicative of myocarditis. Through this strategy, our study contributes to the body of knowledge in cardiovascular magnetic resonance imaging. Future research will focus on combining the different views and types of acquisitions (cine, LGE, T2) in an attempt to extract complementary information and to further enhance the prediction performance. Another future research direction aims at extending this model to differentiate myocarditis from other cardiac conditions, such as ischemic heart disease or cardiomyopathies, which are also commonly encountered in clinical practice.

## Limitations

The dataset’s limited size and notable imbalance (231 myocarditis cases versus 38 controls) present potential limitations to the study’s generalizability, potentially not capturing the full clinical spectrum of myocarditis presentations. The limited data may additionally pose challenges on the multi-frame classification network which has more parameters [31].

This is a single center study with all the annotations being binary labels. For the future we plan to perform (i) a multi-center study with a larger number of samples, (ii) collect multiple expert annotations for each case. This will allow us, amongst others, to perform inter-observer analyses. The CMR views used in Z-Alizadeh dataset are different from the ones available in our dataset. As a result, a direct comparison was not possible.

In this study, our DL model was specifically trained to distinguish between normal and myocarditis cases based on CMRI data. While the model demonstrated high accuracy in differentiating these two groups, it is important to note that this study does not address the detection of myocarditis in the presence of other cardiac conditions. Therefore, the current findings are limited to distinguishing myocarditis from normal heart function, and the generalizability of this model to broader clinical scenarios involving other cardiac pathologies remains to be tested.

The use of k-fold cross-validation in this study may lead to an overestimation of model performance, particularly given the small size of the dataset. This is because, with a limited amount of data, even slight variations in the data splits can significantly influence the results. With the current dataset, we employed the best possible evaluation strategy. However, we acknowledge that a standard training-validation-test split would provide a more robust evaluation framework. We encourage others to build on our work using larger datasets. In the future, we also plan to implement this approach once more data is available.

## Conclusions

In our study, we present a novel deep learning methodology for the detection of myocarditis from CMRI scans. An optimal view for myocarditis detection was identified through rigorous evaluation. Notably, the model exhibited robust learning capabilities even when trained on a small and highly unbalanced dataset, consisting of 231 cases with myocarditis against 38 without myocarditis. Feature analysis revealed that the model’s predictions are predominantly based on characteristics derived from the myocardial region, indicating a targeted approach in identifying pathognomonic patterns indicative of myocarditis.

While all CMR image sequences considered for image-based classification: cine bSSFP, T2 weighted acquisitions, and LGE are clinically relevant and help guide the diagnosis according to [7], our DL classification method obtained the best results on the LGE images acquired in 2 and 4-chamber views. This suggests that the DL network can more reliably use features based on the presence of myocardial scar for the classification.

In the future, we plan to expand the dataset and adopt a standard training-validation-test split to provide a more rigorous evaluation of the model’s performance. This approach will enable us to better assess the performance of the model and address the limitations posed by the current dataset size.

## Data Availability

No datasets were generated or analysed during the current study.
